# 2-[(*E*)-(Morpholin-4-yl­imino)­meth­yl]-6-(morpholin-4-ylmeth­yl)phenol

**DOI:** 10.1107/S160053681302566X

**Published:** 2013-09-21

**Authors:** Mehmet Akkurt, Aliasghar Jarrahpour, Mehdi Mohammadi Chermahini, Mahdi Aberi, Orhan Büyükgüngör

**Affiliations:** a Department of Physics, Faculty of Sciences, Erciyes University, 38039 Kayseri, Turkey; b Department of Chemistry, College of Sciences, Shiraz University, 71454 Shiraz, Iran; c Department of Physics, Faculty of Arts and Sciences, Ondokuz Mayıs University, 55139 Samsun, Turkey

## Abstract

The title compound, C_16_H_23_N_3_O_3_, contains two morpholine rings, each of which adopts a chair conformation. The mol­ecular conformation is stabilized by an intra­molecular O—H⋯N hydrogen bond, leading to a *S*(6) ring. In the crystal, mol­ecules are linked into zigzag chains along the *c*-axis direction by C—H⋯O and C—H⋯π inter­actions.

## Related literature

For background to Schiff bases and their applications, see: Dhar & Taploo (1982 ▶); Nelson *et al.* (2004 ▶); Silva *et al.* (2011 ▶). For ring puckering parameters, see: Cremer & Pople (1975 ▶). For hydrogen-bond motifs, see: Bernstein *et al.* (1995 ▶).
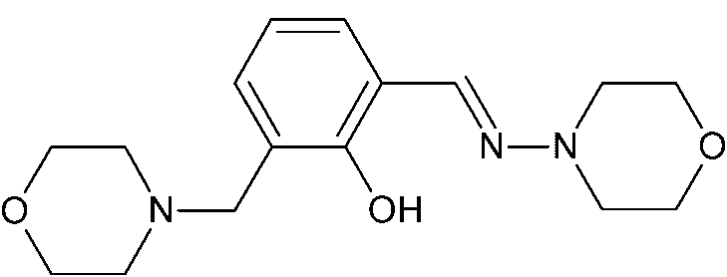


## Experimental

### 

#### Crystal data

C_16_H_23_N_3_O_3_
*M* *_r_* = 305.37
Monoclinic, 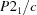

*a* = 9.0074 (6) Å
*b* = 15.7781 (14) Å
*c* = 11.3083 (7) Å
β = 99.052 (5)°
*V* = 1587.1 (2) Å^3^
*Z* = 4
Mo *K*α radiation
μ = 0.09 mm^−1^
*T* = 296 K
0.51 × 0.32 × 0.09 mm

#### Data collection

STOE IPDS 2 diffractometer
Absorption correction: integration (*X-RED32*; Stoe & Cie, 2002 ▶) *T*_min_ = 0.961, *T*_max_ = 0.990
23637 measured reflections
3289 independent reflections
1955 reflections with *I* > 2σ(*I*)
*R*_int_ = 0.089

#### Refinement

*R*[*F* ^2^ > 2σ(*F* ^2^)] = 0.058
*wR*(*F* ^2^) = 0.123
*S* = 1.03
3289 reflections
199 parameters
H-atom parameters constrained
Δρ_max_ = 0.13 e Å^−3^
Δρ_min_ = −0.12 e Å^−3^

### 

Data collection: *X-AREA* (Stoe & Cie, 2002 ▶); cell refinement: *X-AREA*; data reduction: *X-RED32* (Stoe & Cie, 2002 ▶); program(s) used to solve structure: *SHELXS97* (Sheldrick, 2008 ▶); program(s) used to refine structure: *SHELXL97* (Sheldrick, 2008 ▶); molecular graphics: *ORTEP-3 for Windows* (Farrugia, 2012 ▶); software used to prepare material for publication: *WinGX* (Farrugia, 2012 ▶) and *PLATON* (Spek, 2009 ▶).

## Supplementary Material

Crystal structure: contains datablock(s) global, I. DOI: 10.1107/S160053681302566X/tk5257sup1.cif

Structure factors: contains datablock(s) I. DOI: 10.1107/S160053681302566X/tk5257Isup2.hkl

Supplementary material file. DOI: 10.1107/S160053681302566X/tk5257Isup3.cml

Additional supplementary materials:  crystallographic information; 3D view; checkCIF report

## Figures and Tables

**Table 1:** Hydrogen-bond geometry (Å, °) *Cg*3 is the centroid of the C6–C11 benzene ring.

*D*—H⋯*A*	*D*—H	H⋯*A*	*D*⋯*A*	*D*—H⋯*A*
O2—H2⋯N2	0.82	1.91	2.636 (2)	146
C13—H13*A*⋯O3^i^	0.97	2.55	3.416 (3)	149
C4—H4*A*⋯*Cg*3^i^	0.97	2.87	3.646 (3)	138

Symmetry code: (i) 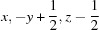.

**Fig. 1.**
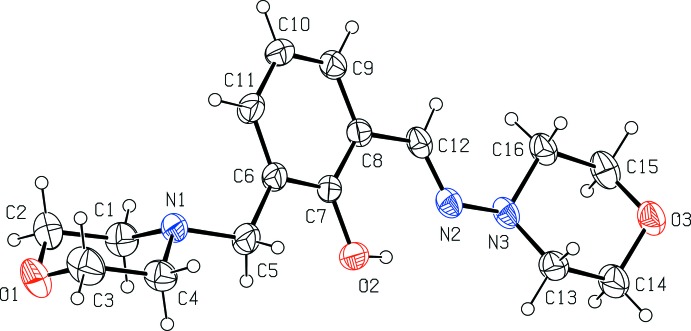
The molecular structure of (I) with the atom-labelling scheme and 30% probability displacement ellipsoids.

**Fig. 2.**
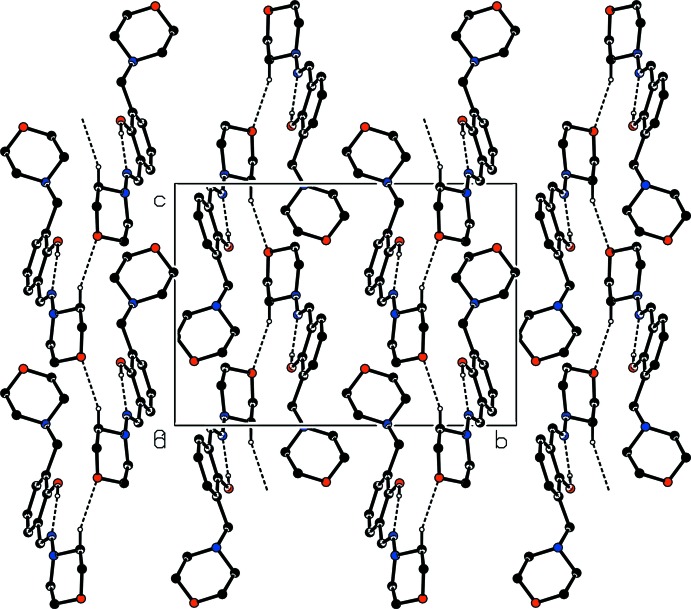
Packing diagram of (I) viewed down the a axis. Hydrogen bonds are indicated by broken lines. H atoms not participating in hydrogen bonding have been omitted for clarity.

## supplementary crystallographic information

### 1. Comment

Schiff bases are formed by the condensation of a primary amine with a carbonyl compound (Dhar & Taploo, 1982). They are widely used for industrial purposes and also exhibit a broad range of biological activities (Silva *et al.*, 2011). The morpholine moiety has been utilized extensively by the pharmaceutical industry in drug design, often because of the improvement in pharmacokinetic properties it can con&shy;fer. The World Drug Index contains well over 100 drugs incorporating this structural feature, including its presence as a side-chain, scaffold, and within fused-ring systems. The biological utility of molecules containing the morpholine moiety is wide-ranging (Nelson *et al.*, 2004). Therefore, Schiff base (I), which has the two morpholine rings, was synthesized and its X-ray structure is reported here.

The two morpholine rings (N1/O1/C1–C4 and N3/O3/C13–C16) of (I), Fig. 1, adopt a chair conformation with puckering parameters (Cremer & Pople, 1975) of Q_T_ = 0.564 (3) Å, θ = 180.0 (2)°, φ = 163 (33)° and Q_T_ = 0.553 (2) Å, θ = 4.6 (2)°, φ = 19 (3)°, respectively. The N1–C5–C6–C7, C5–C6–C7–O2, O2–C7–C8–C12 and C8–C12–N2–N3 torsion angles are -158.0 (2), 0.6 (3), -1.0 (3) and 179.97 (19)°, respectively.

An intramolecular O—H···N hydrogen bond (Table 1) stabilizes the molecular conformation of (I), forming a pseudo six-membered ring with a graph set motif *S*(6) (Bernstein *et al.*, 1995). In the crystal structure, C—H···O hydrogen bonds link the molecules into infinite one-dimensional chains along [001], with a C(4) graph-set motif (Table 1, Fig. 2). Additional C—H···π interactions also assist in the stabilization of the chain.

### 2. Experimental

A mixture of 2-hydroxy-3-(morpholinomethyl)benzaldehyde (1.0 mmol) and morpholin-4-amine (1.0 mmol) was refluxed in EtOH for 4 h. After cooling the solution, the precipitate formed was filtered and recrystallized from ethanol to give yellow crystals in 82% yield. M. pt: 407–409 K. IR (KBr) cm^-1^: 1612 (C=N). ^1^H-NMR (250 MHz, CDCl_3_), δ (p.p.m.): 2.57, 3.15 (CH_2_N, t, 8H, J=5 Hz), 3.66 (CH_2_, s, 2H), 3.75, 3.88 (CH_2_O, t, 8H, J=5 Hz), 6.84 (aromatic H, t, 1H, J=7.5 Hz), 7.23 (aromatic H, d, 2H, J=7.5 Hz), 7.80 (HC=N, s, 1H), 11.71 (OH, s, 1H). ^13^C-NMR (62.9 MHz, CDCl_3_), δ (p.p.m): 51.8, 53.3 (CH_2_N), 57.6 (CH_2_), 66.2, 66.7 (CH_2_O), 118.8–139.1 (aromatic carbons), 156.0 (C=N).

### 3. Refinement

All H atoms were located geometrically with O—H = 0.82 Å, and C—H = 0.93 and 0.97 Å, and refined using a riding model with *U*_iso_(H) = 1.2*U*_eq_(C) for the aromatic- and methylene-H atoms, and *U*_iso_(H) = 1.5*U*_eq_(O) for the hydroxyl-H atom.

### Crystal data

**Table:**

C_16_H_23_N_3_O_3_	*F*(000) = 656
*M**_r_* = 305.37	*D*_x_ = 1.278 Mg m^−^^3^
Monoclinic, *P*2_1_/*c*	Mo *K*α radiation, λ = 0.71073 Å
Hall symbol: -P 2ybc	Cell parameters from 17755 reflections
*a* = 9.0074 (6) Å	θ = 2.2–27.3°
*b* = 15.7781 (14) Å	µ = 0.09 mm^−^^1^
*c* = 11.3083 (7) Å	*T* = 296 K
β = 99.052 (5)°	Shapeless, yellow
*V* = 1587.1 (2) Å^3^	0.51 × 0.32 × 0.09 mm
*Z* = 4	

### Data collection

**Table:**

STOE IPDS 2 diffractometer	3289 independent reflections
Radiation source: sealed X-ray tube, 12 x 0.4 mm long-fine focus	1955 reflections with *I* > 2σ(*I*)
Plane graphite monochromator	*R*_int_ = 0.089
Detector resolution: 6.67 pixels mm^-1^	θ_max_ = 26.5°, θ_min_ = 2.2°
ω–scans	*h* = −11→11
Absorption correction: integration (*X-RED32*; Stoe & Cie, 2002)	*k* = −19→19
*T*_min_ = 0.961, *T*_max_ = 0.990	*l* = −14→14
23637 measured reflections	

### Refinement

**Table:**

Refinement on *F*^2^	0 restraints
Least-squares matrix: full	Hydrogen site location: inferred from neighbouring sites
*R*[*F*^2^ > 2σ(*F*^2^)] = 0.058	H-atom parameters constrained
*wR*(*F*^2^) = 0.123	W = 1/[Σ^2^(*F*O^2^) + (0.0519*P*)^2^] WHERE *P* = (*F*O^2^ + 2*F*C^2^)/3
*S* = 1.03	(Δ/σ)_max_ < 0.001
3289 reflections	Δρ_max_ = 0.13 e Å^−^^3^
199 parameters	Δρ_min_ = −0.12 e Å^−^^3^

### Special details

**Table:**

Geometry. Bond distances, angles *etc*. have been calculated using the rounded fractional coordinates. All su's are estimated from the variances of the (full) variance-covariance matrix. The cell e.s.d.'s are taken into account in the estimation of distances, angles and torsion angles
Refinement. Refinement on *F*^2^ for ALL reflections except those flagged by the user for potential systematic errors. Weighted *R*-factors *wR* and all goodnesses of fit *S* are based on *F*^2^, conventional *R*-factors *R* are based on *F*, with *F* set to zero for negative *F*^2^. The observed criterion of *F*^2^ > σ(*F*^2^) is used only for calculating -*R*-factor-obs *etc*. and is not relevant to the choice of reflections for refinement. *R*-factors based on *F*^2^ are statistically about twice as large as those based on *F*, and *R*-factors based on ALL data will be even larger.

### Fractional atomic coordinates and isotropic or equivalent isotropic displacement parameters (Å^2^)

**Table:**

	*x*	*y*	*z*	*U*_iso_*/*U*_eq_	
O1	0.7886 (2)	0.05694 (15)	0.26574 (16)	0.0912 (9)	
O2	0.46117 (17)	0.15800 (11)	0.73906 (12)	0.0586 (5)	
O3	0.15802 (19)	0.22570 (11)	1.21636 (13)	0.0659 (6)	
N1	0.7555 (2)	0.12192 (11)	0.49469 (14)	0.0497 (6)	
N2	0.3995 (2)	0.13953 (12)	0.95782 (15)	0.0500 (6)	
N3	0.2980 (2)	0.14585 (12)	1.03797 (14)	0.0514 (6)	
C1	0.7095 (3)	0.03684 (16)	0.4572 (2)	0.0669 (9)	
C2	0.8012 (4)	0.00352 (19)	0.3670 (2)	0.0838 (11)	
C3	0.8362 (4)	0.1393 (2)	0.3018 (2)	0.0859 (13)	
C4	0.7453 (3)	0.17607 (16)	0.38952 (19)	0.0614 (8)	
C5	0.6625 (3)	0.15608 (18)	0.57868 (19)	0.0667 (9)	
C6	0.7055 (3)	0.12328 (14)	0.70585 (18)	0.0523 (8)	
C7	0.5991 (2)	0.12627 (13)	0.78287 (18)	0.0467 (7)	
C8	0.6357 (2)	0.09994 (13)	0.90283 (18)	0.0452 (7)	
C9	0.7809 (3)	0.07110 (14)	0.9425 (2)	0.0562 (8)	
C10	0.8869 (3)	0.06887 (16)	0.8678 (2)	0.0629 (9)	
C11	0.8489 (3)	0.09507 (15)	0.7501 (2)	0.0600 (9)	
C12	0.5296 (3)	0.10624 (14)	0.98800 (18)	0.0501 (8)	
C13	0.2093 (3)	0.22298 (16)	1.01350 (19)	0.0584 (9)	
C14	0.0910 (3)	0.22850 (19)	1.0939 (2)	0.0691 (10)	
C15	0.2348 (3)	0.14835 (18)	1.2391 (2)	0.0707 (10)	
C16	0.3593 (3)	0.13650 (15)	1.16513 (18)	0.0566 (8)	
H1A	0.60400	0.03710	0.42230	0.0800*	
H1B	0.72160	−0.00030	0.52650	0.0800*	
H2	0.40790	0.15670	0.79150	0.0880*	
H2A	0.90580	−0.00040	0.40360	0.1010*	
H2B	0.76680	−0.05290	0.34220	0.1010*	
H3A	0.82770	0.17570	0.23190	0.1030*	
H3B	0.94120	0.13770	0.33800	0.1030*	
H4A	0.78230	0.23230	0.41320	0.0740*	
H4B	0.64110	0.18110	0.35220	0.0740*	
H5A	0.55830	0.14180	0.55030	0.0800*	
H5B	0.67080	0.21740	0.57950	0.0800*	
H9	0.80660	0.05290	1.02130	0.0670*	
H10	0.98380	0.04990	0.89600	0.0750*	
H11	0.92120	0.09370	0.69980	0.0720*	
H12	0.55720	0.08570	1.06540	0.0600*	
H13A	0.16130	0.22320	0.93050	0.0700*	
H13B	0.27480	0.27200	1.02640	0.0700*	
H14A	0.03510	0.28090	1.07820	0.0830*	
H14B	0.02110	0.18170	1.07680	0.0830*	
H15A	0.16360	0.10220	1.22240	0.0850*	
H15B	0.27740	0.14560	1.32330	0.0850*	
H16A	0.43750	0.17840	1.18800	0.0680*	
H16B	0.40340	0.08060	1.17960	0.0680*	

### Atomic displacement parameters (Å^2^)

**Table:**

	*U*^11^	*U*^22^	*U*^33^	*U*^12^	*U*^13^	*U*^23^
O1	0.1117 (17)	0.1190 (17)	0.0446 (10)	0.0059 (13)	0.0173 (10)	−0.0190 (11)
O2	0.0528 (9)	0.0838 (11)	0.0411 (8)	0.0177 (8)	0.0129 (7)	0.0064 (8)
O3	0.0724 (12)	0.0822 (12)	0.0457 (9)	0.0009 (9)	0.0178 (8)	−0.0106 (8)
N1	0.0550 (11)	0.0584 (12)	0.0378 (9)	0.0049 (9)	0.0141 (8)	0.0022 (8)
N2	0.0553 (12)	0.0586 (11)	0.0379 (9)	−0.0038 (9)	0.0128 (8)	−0.0032 (8)
N3	0.0578 (11)	0.0657 (12)	0.0339 (9)	−0.0087 (10)	0.0168 (8)	−0.0046 (8)
C1	0.0723 (17)	0.0674 (16)	0.0628 (15)	−0.0028 (13)	0.0164 (13)	0.0056 (13)
C2	0.101 (2)	0.085 (2)	0.0652 (17)	0.0133 (17)	0.0121 (16)	−0.0203 (16)
C3	0.086 (2)	0.122 (3)	0.0555 (16)	−0.0115 (19)	0.0288 (15)	0.0107 (17)
C4	0.0664 (15)	0.0706 (16)	0.0480 (13)	−0.0028 (13)	0.0118 (11)	0.0105 (12)
C5	0.0764 (17)	0.0838 (17)	0.0434 (13)	0.0302 (14)	0.0202 (12)	0.0073 (12)
C6	0.0625 (15)	0.0575 (14)	0.0383 (11)	0.0173 (11)	0.0119 (10)	0.0016 (10)
C7	0.0533 (14)	0.0458 (12)	0.0409 (11)	0.0097 (10)	0.0070 (10)	−0.0036 (9)
C8	0.0555 (14)	0.0405 (11)	0.0396 (11)	0.0037 (10)	0.0075 (10)	−0.0008 (9)
C9	0.0722 (16)	0.0564 (14)	0.0390 (12)	0.0141 (12)	0.0053 (11)	0.0014 (10)
C10	0.0619 (16)	0.0742 (16)	0.0509 (14)	0.0248 (13)	0.0036 (12)	0.0025 (12)
C11	0.0608 (16)	0.0727 (16)	0.0494 (13)	0.0222 (12)	0.0177 (11)	0.0032 (12)
C12	0.0702 (17)	0.0460 (12)	0.0352 (11)	−0.0021 (11)	0.0122 (11)	−0.0006 (9)
C13	0.0563 (15)	0.0766 (17)	0.0433 (12)	0.0045 (12)	0.0110 (11)	0.0012 (12)
C14	0.0611 (16)	0.099 (2)	0.0503 (14)	−0.0016 (14)	0.0181 (12)	−0.0086 (14)
C15	0.094 (2)	0.0805 (18)	0.0419 (13)	−0.0078 (16)	0.0245 (13)	−0.0040 (13)
C16	0.0771 (16)	0.0586 (15)	0.0355 (11)	0.0008 (12)	0.0129 (11)	0.0013 (10)

### Geometric parameters (Å, º)

**Table:**

O1—C2	1.412 (3)	C15—C16	1.512 (4)
O1—C3	1.409 (4)	C1—H1A	0.9700
O2—C7	1.359 (2)	C1—H1B	0.9700
O3—C14	1.422 (3)	C2—H2A	0.9700
O3—C15	1.406 (3)	C2—H2B	0.9700
O2—H2	0.8200	C3—H3A	0.9700
N1—C1	1.449 (3)	C3—H3B	0.9700
N1—C5	1.465 (3)	C4—H4A	0.9700
N1—C4	1.456 (3)	C4—H4B	0.9700
N2—N3	1.389 (2)	C5—H5A	0.9700
N2—C12	1.281 (3)	C5—H5B	0.9700
N3—C16	1.464 (3)	C9—H9	0.9300
N3—C13	1.458 (3)	C10—H10	0.9300
C1—C2	1.505 (4)	C11—H11	0.9300
C3—C4	1.499 (4)	C12—H12	0.9300
C5—C6	1.520 (3)	C13—H13A	0.9700
C6—C7	1.394 (3)	C13—H13B	0.9700
C6—C11	1.383 (4)	C14—H14A	0.9700
C7—C8	1.407 (3)	C14—H14B	0.9700
C8—C12	1.463 (3)	C15—H15A	0.9700
C8—C9	1.391 (3)	C15—H15B	0.9700
C9—C10	1.371 (4)	C16—H16A	0.9700
C10—C11	1.384 (3)	C16—H16B	0.9700
C13—C14	1.508 (4)		
			
C2—O1—C3	109.51 (19)	O1—C3—H3B	109.00
C14—O3—C15	109.05 (18)	C4—C3—H3A	109.00
C7—O2—H2	109.00	C4—C3—H3B	109.00
C1—N1—C5	111.37 (19)	H3A—C3—H3B	108.00
C4—N1—C5	110.18 (18)	N1—C4—H4A	110.00
C1—N1—C4	109.06 (17)	N1—C4—H4B	110.00
N3—N2—C12	121.63 (17)	C3—C4—H4A	110.00
N2—N3—C16	116.65 (18)	C3—C4—H4B	110.00
C13—N3—C16	112.40 (17)	H4A—C4—H4B	108.00
N2—N3—C13	109.48 (17)	N1—C5—H5A	109.00
N1—C1—C2	111.1 (2)	N1—C5—H5B	109.00
O1—C2—C1	111.0 (2)	C6—C5—H5A	109.00
O1—C3—C4	112.1 (3)	C6—C5—H5B	109.00
N1—C4—C3	110.0 (2)	H5A—C5—H5B	108.00
N1—C5—C6	113.7 (2)	C8—C9—H9	119.00
C5—C6—C7	118.8 (2)	C10—C9—H9	119.00
C5—C6—C11	122.4 (2)	C9—C10—H10	120.00
C7—C6—C11	118.66 (19)	C11—C10—H10	120.00
O2—C7—C8	121.49 (17)	C6—C11—H11	119.00
C6—C7—C8	120.84 (18)	C10—C11—H11	119.00
O2—C7—C6	117.64 (18)	N2—C12—H12	119.00
C7—C8—C9	118.25 (18)	C8—C12—H12	119.00
C9—C8—C12	119.24 (19)	N3—C13—H13A	110.00
C7—C8—C12	122.43 (18)	N3—C13—H13B	110.00
C8—C9—C10	121.4 (2)	C14—C13—H13A	109.00
C9—C10—C11	119.6 (2)	C14—C13—H13B	110.00
C6—C11—C10	121.3 (2)	H13A—C13—H13B	108.00
N2—C12—C8	121.17 (18)	O3—C14—H14A	109.00
N3—C13—C14	110.6 (2)	O3—C14—H14B	109.00
O3—C14—C13	110.7 (2)	C13—C14—H14A	110.00
O3—C15—C16	113.1 (2)	C13—C14—H14B	110.00
N3—C16—C15	109.4 (2)	H14A—C14—H14B	108.00
N1—C1—H1A	109.00	O3—C15—H15A	109.00
N1—C1—H1B	109.00	O3—C15—H15B	109.00
C2—C1—H1A	109.00	C16—C15—H15A	109.00
C2—C1—H1B	109.00	C16—C15—H15B	109.00
H1A—C1—H1B	108.00	H15A—C15—H15B	108.00
O1—C2—H2A	109.00	N3—C16—H16A	110.00
O1—C2—H2B	109.00	N3—C16—H16B	110.00
C1—C2—H2A	109.00	C15—C16—H16A	110.00
C1—C2—H2B	109.00	C15—C16—H16B	110.00
H2A—C2—H2B	108.00	H16A—C16—H16B	108.00
O1—C3—H3A	109.00		
			
C2—O1—C3—C4	−59.1 (3)	N1—C5—C6—C7	−158.0 (2)
C3—O1—C2—C1	58.2 (3)	C5—C6—C11—C10	177.2 (2)
C14—O3—C15—C16	60.8 (3)	C11—C6—C7—O2	176.9 (2)
C15—O3—C14—C13	−61.0 (3)	C5—C6—C7—O2	0.6 (3)
C1—N1—C4—C3	−55.5 (3)	C7—C6—C11—C10	1.0 (3)
C4—N1—C1—C2	55.8 (3)	C11—C6—C7—C8	−1.0 (3)
C5—N1—C4—C3	−178.0 (2)	C5—C6—C7—C8	−177.3 (2)
C1—N1—C5—C6	78.8 (2)	O2—C7—C8—C9	−177.62 (19)
C4—N1—C5—C6	−160.1 (2)	O2—C7—C8—C12	−1.0 (3)
C5—N1—C1—C2	177.6 (2)	C6—C7—C8—C12	176.8 (2)
C12—N2—N3—C16	17.4 (3)	C6—C7—C8—C9	0.2 (3)
C12—N2—N3—C13	146.5 (2)	C7—C8—C12—N2	−2.9 (3)
N3—N2—C12—C8	−179.97 (19)	C12—C8—C9—C10	−176.1 (2)
C13—N3—C16—C15	50.3 (3)	C7—C8—C9—C10	0.6 (3)
N2—N3—C13—C14	176.21 (18)	C9—C8—C12—N2	173.7 (2)
C16—N3—C13—C14	−52.5 (3)	C8—C9—C10—C11	−0.6 (4)
N2—N3—C16—C15	177.92 (19)	C9—C10—C11—C6	−0.3 (4)
N1—C1—C2—O1	−58.0 (3)	N3—C13—C14—O3	57.5 (3)
O1—C3—C4—N1	58.4 (3)	O3—C15—C16—N3	−55.1 (3)
N1—C5—C6—C11	25.8 (3)		

### Hydrogen-bond geometry (Å, º)

Cg3 is the centroid of the C6–C11 benzene ring.

**Table:**

*D*—H···*A*	*D*—H	H···*A*	*D*···*A*	*D*—H···*A*
O2—H2···N2	0.82	1.91	2.636 (2)	146
C13—H13*A*···O3^i^	0.97	2.55	3.416 (3)	149
C4—H4*A*···*Cg*3^i^	0.97	2.87	3.646 (3)	138

Symmetry code: (i) *x*, −*y*+1/2, *z*−1/2.

## References

[bb1] Bernstein, J., Davis, R. E., Shimoni, L. & Chang, N.-L. (1995). *Angew. Chem. Int. Ed. Engl.* **34**, 1555–1573.

[bb2] Cremer, D. & Pople, J. A. (1975). *J. Am. Chem. Soc.* **97**, 1354–1358.

[bb3] Dhar, D. N. & Taploo, C. L. (1982). *J. Sci. Ind. Res.* **41**, 501–506.

[bb4] Farrugia, L. J. (2012). *J. Appl. Cryst.* **45**, 849–854.

[bb5] Nelson, T. D., Rosen, J. D., Brands, K. M. J., Craig, B., Huffman, M. A. & McNamara, J. M. (2004). *Tetrahedron Lett.* **45**, 8917–8920.

[bb6] Sheldrick, G. M. (2008). *Acta Cryst.* A**64**, 112–122. 10.1107/S0108767307043930 18156677

[bb7] Silva, C. M., da Silva, D. L., Modolo, L. V., Alves, R. B., de Resende, M. A., Martins, C. V. B. & de Fatima, A. (2011). *J. Adv. Res.* **2**, 1–8.

[bb8] Spek, A. L. (2009). *Acta Cryst.* D**65**, 148–155. 10.1107/S090744490804362X PMC2631630 19171970

[bb9] Stoe & Cie (2002). *X-AREA* and *X-RED32* Stoe & Cie, Darmstadt, Germany.

